# Brief report: can COVID-19 infection trigger rheumatoid arthritis-associated autoimmunity in individuals at risk for the disease? A nested cohort study

**DOI:** 10.3389/fmed.2023.1201425

**Published:** 2023-07-07

**Authors:** Celine Lamacchia, Benoit Gilbert, Olivia Studer, Kim Lauper, Axel Finckh

**Affiliations:** ^1^Division of Rheumatology, Geneva University Hospitals and Faculty of Medicine, University of Geneva, Geneva, Switzerland; ^2^The Geneva Center For Inflammation Research, University of Geneva, Geneva, Switzerland

**Keywords:** rheumatoid arthritis, SARS-CoV-2, COVID-19, first-degree relatives, RA-associated autoimmunity, pre-clinical stages of RA

## Abstract

**Objectives:**

To investigate the association between severe acute respiratory syndrome-associated coronavirus-2 (SARS-CoV-2) infection and subsequent development of autoimmunity or pre-clinical manifestations associated with rheumatoid arthritis (RA) in at risk population.

**Methods:**

This is a nested study within a prospective cohort of first-degree relatives of RA patients (RA-FDR). Participants are tested for RA-associated autoantibodies (anti-citrullinated peptide antibodies (ACPA)/rheumatoid factor (RF)) and clinical signs and symptoms suggestive of early disease. SARS-CoV-2 infections were self-reported between March 2020 and January 2023. All individuals with a pre-pandemic (sample 1) and a post-pandemic sample (sample 2) were included in the analysis. The exposure of interest was self-reported SARS-CoV-2 infection. The primary outcome was a clinically significant change in RA-associated autoantibody serum titers. Secondary outcomes included: becoming seropositive, becoming symptomatic, developing classifiable RA.

**Results:**

Among 168 RA-FDRs, 109 reported a SARS-CoV-2 infection between sample 1 and sample 2. During this period, 2 RA-FDRs (1.2%) became anti-CCP2 seropositive, none became anti-CCP3 IgG positive, 6 (3.6%) became RF IgM seropositive, 1 became (0.6%) RF IgA seropositive, 19 (11.3%) became symptomatic and none developed classifiable RA. SARS-CoV-2 infection was not significantly associated with increases in RA autoantibody titers or with secondary outcomes.

**Conclusion:**

We could not detect an association between SARS-CoV-2 infection and subsequent development of RA-associated autoimmunity, nor signs or symptoms of RA in an at risk population. These findings do not support the hypothesis that SARS-CoV-2 infections triggers the immune onset of RA.

## Highlights

- Preliminary case reports have suggested that acute SARS-CoV-2 infection could trigger rheumatoid arthritis (RA) onset.
- In order to assess the potential for SARS-CoV-2 to trigger RA disease onset, this study analyzed prospectively the systemic autoimmunity and preclinical manifestations associated with RA in a cohort of individuals at risk for the disease during the SARS-CoV-2 pandemic.
- The development of systemic autoimmunity associated with RA and the development of clinical signs and symptoms of the disease remained rare and did not appear to increase after a COVID-19. We found no evidence that an acute SARS-CoV-2 infections triggers the immune onset of RA.

## Introduction

Coronavirus disease 2019 (COVID-19) is caused by an infection with the severe acute respiratory syndrome-associated coronavirus-2 (SARS-CoV-2). In a subset of patients, COVID-19 can lead to severe inflammation and dysregulation of the immune system, which has been suggested as a potential trigger for autoimmune responses. Several studies have reported autoantibodies in the sera of COVID-19 patients and the development of autoimmune diseases after SARS-CoV-2 infection (1, 2). Various mechanisms have been proposed to explain these observations, such as molecular mimicry, bystander activation, epitope spreading, viral persistence and/or formation of neutrophil extracellular traps (3).

Arthritic manifestations following SARS-CoV-2 infection have also been reported, including a few cases of reactive arthritis or post-viral arthritis (4–9). Perrot et al. reported a flaring of seropositive rheumatoid arthritis (RA) with a large increase in anti-citrullinated protein antibody (ACPA) titers (3× the norm for anti-CCP2) after SARS-CoV-2 infection (10). Another case of ACPA-positive symmetric peripheral polyarthritis has been described after SARS-CoV-2 infection, but without information on ACPA levels before the onset of symptomatic arthritis (11). In addition, in a Korean study including 24′117 patients, the authors observed that endemic human coronaviruses are associated with an increased risk of developing RA (12). The main question raised by this literature is whether SARS-CoV-2 infections can trigger the onset of RA by inducing or increasing RA-associated autoimmunity in susceptible individuals. Currently, no answer can be provided due to the limited number of case-reports and the absence of prospective cohort studies in at risk populations.

The aim of the present study was to investigate a potential link between SARS-CoV-2 infection and the development of autoimmunity and pre-clinical manifestations associated with RA in a cohort of individuals at risk for RA, namely first-degree relatives of RA patients (RA-FDR).

## Methods

### Study population

This study was performed in an ongoing cohort of first-degree relatives of RA patients (RA-FDRs) (the SCREEN-RA cohort) (13). A detailed description of this cohort is available elsewhere (13), but briefly RA-FDRs without rheumatic disease at baseline are enrolled and followed up yearly, fill out a number of epidemiologic questionnaires, are examined by a health professional and provide serum samples at each visit. The data for this analysis was extracted on 2023-01-09. Our data analysis was approved by the local Geneva Ethics Committee (protocol 2021-02005). All participants gave informed consent before enrolment in accordance with the Declaration of Helsinki.

### Study design and exposure of interest

This is a nested study within the prospective SCREEN-RA cohort. We first considered RA-FDRs for which serum samples were available before and after the COVID-19 outbreak. Specifically, we selected participants who provided a pre-pandemic sample (sample 1), which was collected prior to March 2020, and a per-post pandemic sample (sample 2), which was collected after March 2020 and before January 2023. If several pre-pandemic samples were available, we chose the assessment closest to March 2020, and only blood samples after reporting COVID-19 status were taken into account for sample 2. Whenever multiple post-pandemic samples were available, we prioritized the later assessments for our analysis.

In parallel, a questionnaire related to SARS-CoV-2 infection was implemented in the yearly follow-up questionnaire and administered between March 2020 and January 2023. The self-reported SARS-CoV-2 questionnaire included SARS-CoV-2 diagnostic test results (Positive, Negative, or Unknown), date and the type of test used (Nasopharyngeal sampling for a PCR test or blood sampling for the detection of antibodies against SARS-Cov-2 (anti-spike subunit 1 (S1) assay) or Unknown), presence of symptoms suggestive of SARS-CoV-2 infection (runny nose/fever/headache/sore throat/cough/shortness of breath/muscle or body aches/fatigue/diarrhoea/change in smell or taste), hospitalization and vaccination dates.

**Figure 1:**
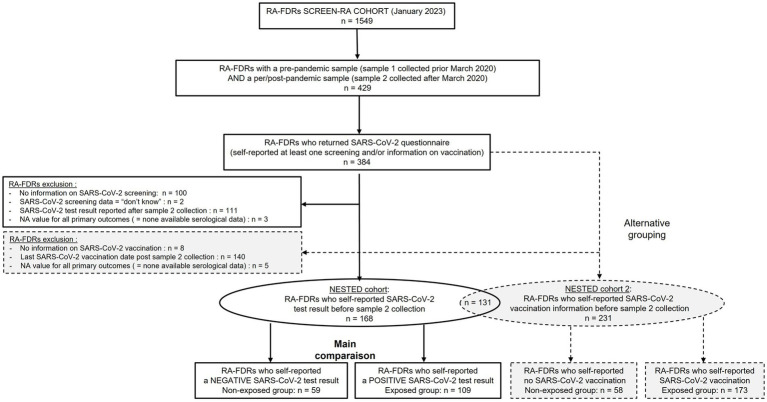
Study flow chart. RA-FDR, first degree relatives of RA patients.

The primary exposure of interest was self-reported SARS-CoV-2 infection. As an alternative grouping, we also performed comparison between non-vaccinated and vaccinated subjects. Only RA-FDR who provided information on SARS-Cov-2 (SARS-CoV-2 test result or SARS-CoV-2 vaccination date(s)) before the collection of sample 2 were analyzed. The study flow chart is displayed in Figure 1.

### Autoantibodies and symptoms associated with RA

The following enzyme-linked immunosorbent assays (ELISAs) were used to define anti-cyclic citrullinated peptide 2 (anti-CCP2), anti-CCP3 IgG, rheumatoid factor (RF) IgM and RF IgA positivity, respectively, according to the manufacturers’ cut-off values (1× ≥ the upper limit of normal (ULN)): CCPlus^®^ Immunoscan (Eurodiagnostica; ≥25 U/mL), QUANTA Lite^®^ CCP3 IgG (Inova Diagnostics; ≥20 U/mL), QUANTA Lite RF IgM and IgA^®^ ELISAs (Inova Diagnostics; ≥6 U/mL). RA-associated symptoms were defined either by the presence of a clinically suspect arthralgia (CSA) or the presence of an inflammatory arthritis defined by having at least one swollen joint at physical examination (or self-reported by the participant in case of missing data). For the definition of CSA, we used the EULAR instrument, with four or more out the seven criteria (symptom duration < 1 year, symptoms in metacarpophalangeal joints, morning stiffness duration ≥60 min, most severe symptoms in early morning, being RA-FDR, difficulty to making a fist, and positive squeeze test of metacarpophalangeal joints) (14).

### Primary outcome

The primary outcome of this analysis was a substantial change in RA-associated autoantibody serum titers between sample 1 and sample 2. An increase beyond at least 1× the upper limit of the norm (≥1× ULN) for the respective ELISA test was considered biologically relevant. In this case, participants would have to be positive for the auto-antibody at the time of sample 2, while having been negative or weakly positive at the time of sample 1. The outcome is depicted in tables by the following variables: “Positive increase of anti-CCP2 ratio,” “Positive increase of anti-CCP3 IgG ratio,” “Positive increase of RF IgM ratio” and “Positive increase of RF IgA ratio.”

### Secondary outcomes

The secondary outcomes of this analysis were: becoming newly anti-CCP2, anti-CCP3 IgG, RF IgM or RF IgA positive between sample 1 and sample 2. This binary outcome was coded 1 if the patient was negative at the time of sample 1 and positive at the time of sample 2 using the cut-offs of the respective ELISA tests, and was coded 0 otherwise (stayed positive, stayed negative or switched from positive to negative). Becoming symptomatic (i.e., becoming positive for CSA or to develop at least one swollen joint) and developing classifiable RA between sample 1 and sample 2 were two other secondary outcomes. The development of classifiable RA was defined by a board-certified rheumatologist.

### Statistical analysis

Continuous variables were presented as means ± standard deviations (SD), or as medians and interquartile ranges (IQR) when appropriate. Categorical variables were compared between exposed and non-exposed groups using the chi square (Chi2) test or Fisher’s exact test if small samples size (i.e., less than 5 observations). Continuous variables were compared using *t*-tests, or Wilcoxon tests in case of non-normal distribution. Crude incidence rate (IR) and crude relative risk (RR) was used as indicator for measuring the strength of the association between the exposure of interest and each outcome in a complete case analysis. All statistical tests were two-sided, and *p*-values of 0.05 or less were considered statistically significant. All analyses were performed with R software (V.4.0.4) using package *tableone*.

### Patient and public involvement statement

This research was done without patient involvement. Patients were not invited to comment on the study design and were not consulted to develop patient relevant outcomes or interpret the results. Patients were not invited to contribute to the writing or editing of this document for readability or accuracy.

## Results

Patient-characteristics and the serological status of all RA-FDR (*n* = 168) who had a pre-pandemic (sample 1) and a per/post-pandemic (sample 2) samples and who reported a SARS-Cov-2 test result before the sample 2 collection date are presented in Table 1. Most participants were women (79%), and the mean age was 52 years-old at sample 2. In the overall population, the baseline prevalence of anti-CCP2, anti-CCP3 IgG, RF IgM and RF IgA was 3.6%, 1.8%, 14.3%, and 3.0%, respectively. RA-associated symptoms were present in 27 individuals (16%) and one participant developed incident RA. During this period of time, 2 (1.2%) RA-FDRs became anti-CCP2 positive, none developed anti-CCP3 IgG positivity, 6 (3.6%) became RF IgM positive and 1 (0.6%) RF IgA positive (Table 2). We observed a substantial increase of anti-CCP2 in 2.4% (*n* = 4), RF IgM in 4.8% (*n* = 8), and RF IgA in 1.2% (*n* = 2) of RA-FDR. During the same period, 19 (11.3%) RA-FDR developed RA-associated symptoms and none RA-FDR developed classifiable RA.

**Table 1:** Socio-demographic characteristics and serological status of RA-FDRs with self-reported data associated with Sars-CoV-2 infection.

		RA-FDRs with no self-reported Sars-CoV-2 infection	RA-FDRs with self-reported Sars-CoV-2 infection	*p*	Missing (%)
RA-FDRs (*n*)		59	109		
Gender = female (*n* (%))		48 (81.4)	85 (78.0)	0.75	
Age (years; mean (SD))		55.3 (11.7)	50.7 (12.2)	0.02	
BMI (mean (SD))		25.2 (5.4)	23.9 (3.5)	0.05	
Shared epitope (2 copies; *n* (%))		16 (27.1)	16 (14.7)	0.15	
Anti-CCP2 positive (*n* (%))		5 (8.5)	1 (0.9)	0.02	13 (7.7)
Anti-CCP3 IgG positive (*n* (%))		2 (3.4)	1 (0.9)	0.34	23 (13.7)
RF IgM positive (*n* (%))		12 (20.3)	12 (11.0)	0.10	13 (7.7)
RF IgA positive (*n* (%))		2 (3.4)	3 (2.8)	0.30	13 (7.7)
RA-associated symptoms (*n* (%))		13 (22.2)	14 (12.8)	0.18	1 (0.6)
Incident RA (*n* (%))		0 (0)	1 (0.9)	1.00	
Self-reported data	Sars-CoV-2 test			0.15	1 (0.6)
	- PCR test (*n* (%))	55 (93.2)	88 (80.7)		
	- Anti-S1 serology (*n* (%))	3 (5.1)	18 (16.5)		
	Sars-CoV-2-associated symptoms (*n* (%))	0 (0.0)	42 (38.5)	<0.01	59 (35.1)
	Sars-CoV-2 vaccination (*n* (%))	50 (84.7)	91 (83.5)	0.18	4 (2.4)
	Sars-CoV-2-associated hospitalisation (*n* (%))	0 (0.0)	4 (3.7)	<0.01	120 (71.4)

All variables were defined at sample 2 (per/post-pandemic sample) collection. Anti-CCP2, anti-CCP3 IgG, RF IgM, and RF IgA positivity: 1 time upper limit of normal (ULN); RA-associated symptoms: clinically suspected arthralgia (CSA) or presence of at least one swollen joint. RA, rheumatoid arthritis; RF, rheumatoid factor; anti-CCP, anti-cyclic citrullinated peptide; FDR, first-degree relative; PCR, polymerase chain reaction; S1, spike subunit 1.

**Table 2:** Evolution of RA-associated autoantibodies and symptoms in a nested RA-FDR cohort during SARS-CoV-2 pandemic.

	RA-FDRs with no self-reported Sars-CoV-2 infection	Missing (%)	RA-FDRs with self-reported Sars-CoV-2 infection	Missing (%)	Crude RR (95% CI)
RA-FDRs (*n*)	59		109		
Primary outcomes					
Positive increase of anti-CCP2 ratio (≥1x ULN) (*n* (%))	3 (5.1)	2 (3.4)	1 (0.9)	13 (11.9)	0.20 (0.02–1.86)
Positive increase of anti-CCP3 IgG ratio (≥1x ULN) (*n* (%))	0 (0.0)	42 (71.2)	0 (0.0)	68 (62.4)	1.00
Positive increase of RF IgM ratio (≥1x ULN) (*n* (%))	5 (8.5)	17 (28.8)	3 (2.8)	41 (37.6)	0.37 (0.09–1.47)
Positive increase of RF IgA ratio (≥1x ULN) (*n* (%))	1 (1.7)	17 (28.8)	1 (0.9)	41 (37.6)	0.62 (0.04–9.61)
Secondary outcomes					
Becoming CCP2 positive (*n* (%))	1 (1.7)	2 (3.4)	1 (0.9)	13 (11.9)	0.60 (0.04–9.41)
Becoming CCP3 positive (*n* (%))	0 (0.0)	42 (71.2)	0 (0.0)	68 (62.4)	1.00
Becoming RF IgM positive (*n* (%))	5 (8.5)	17 (28.8)	1 (0.9)	41 (37.6)	0.12 (0.02–1.03)
Becoming RF IgA positive (*n* (%))	0 (0.0)	17 (28.8)	1 (0.9)	41 (37.6)	NA
Developing RA-associated symptoms (*n* (%))	11 (18.6)	1 (1.7)	8 (7.3)	0 (0.0)	0.39 (0.15–0.91)
RA development (*n* (%))	0 (0.0)	/	0 (0.0)	/	1.00

All variables were defined at sample 2 (per/post-pandemic sample) collection. Positive increase of anti-CCP2, anti-CCP3 IgG, RF IgM, and RF IgA ratio: an increase beyond at least 1× the upper limit of the norm (≥1× ULN) between sample 2 and sample 1 (pre-pandemic sample); RA-associated symptoms: clinically suspected arthralgia (CSA) or presence of at least one swollen joint. RA, rheumatoid arthritis; RF, rheumatoid factor; anti-CCP, anti-cyclic citrullinated peptide; FDR, first-degree relative; NA, not applicable; RR, relative risk; CI, confidence intervals. A complete case analysis was used to determine crude RR.

Sixty-five % (*n* = 109) of these participants reported a SARS-CoV-2 infection between sample 1 and sample 2. The median time difference between both samples was 3.8 years [3.0–8.1] and 5.7 years [3.1–8.9] in the non-infected and infected population, respectively (value of *p* = 0.13). In the infected population, 38.5% (*n* = 45) had self-reported symptoms related to SARS-CoV-2 and 3.7% (*n* = 4) were hospitalized after severe infection (Table 1). The proportion of RA-FDRs in whom serum ACPA or RF auto-antibodies increased substantially was not significantly different between the group reporting SARS-CoV-2 infection and the group reporting no SARS-CoV-2 infection (Table 2). No significant difference related to the proportion of individuals (1) becoming positive for at least one RA-associated autoantibodies, (2) developing RA-associated symptoms or (3) developing classifiable RA, was observed between SARS-CoV-2 infected and not infected RA-FDRs (Table 2). Supplementary Table S3 showed crude incidence rates (IR) per 1,000 patients-year for each outcome in exposed and non-exposed groups. The absence of associations was determined using crude risk ratios (RR) (Table 2).

Similar results were observed when comparing vaccinated RA-FDR group (*n* = 173) with unvaccinated RA-FDR group (*n* = 58) (Supplementary Table S1, S2, and S4; Figure 1).

## Discussion

The SARS-CoV-2 infection has been proposed as a potential trigger for the development of autoimmunity and musculoskeletal symptoms associated with rheumatic disorders (7, 8). We assessed ACPA and RF autoantibodies and clinically suspect arthralgias in a cohort of individuals at risk for RA exposed to SARS-CoV-2. We found no impact of SARS-CoV-2 infection on signs or symptoms associated with RA disease development in this population particularly at risk.

It is speculated that severe COVID-19 could trigger a breach of tolerance to citrullinated proteins and induce the formation of ACPA, known to be pathogenic in RA (15, 16). In our study, four RA-FDRs who reported having SARS-CoV-2 infection were hospitalized. None of them had subsequent increased levels of anti-CCP2 or anti-CCP3 IgG antibodies in their post-pandemic serum samples. In addition, none of them produced RFs or developed RA-associated symptoms during their follow-up period. The rest of included participants self-reported mild to moderate COVID-19 symptoms, probably limiting the potential effect of SARS-CoV-2 infection on RA disease development. However, longer-term follow-up would be needed to rule out association between long COVID-19 and RA development.

Flares or new-onset of autoimmune disorders have also been reported soon after SARS-CoV-2 mRNA vaccination (17, 18), including for RA. Interestingly, Yonezawa et al. reported a new-onset of seropositive RA following SARS-CoV-2 mRNA vaccination in a patient with a seronegative status before vaccination (19). Still, we found no evidence of a significant impact of SARS-CoV-2 vaccination on the autoimmune status of this at-risk participants. The mechanisms underlying RA-associated autoimmunity after vaccination are not clear and we cannot exclude the possibility that the onset of RA development in this case report with regard to vaccination was coincidental.

Regional variations in SARS-CoV-2 strains, disease severity and study population genetics could explain disparities between our study and previous reports. Indeed, the genetic diversity and evolving nature of the virus, combined with the individual characteristics of the study population, may have implications on the immune response and the subsequent development of autoimmune manifestations, including RA-related outcomes.

The strength of this longitudinal study is a representative sample of at-risk individuals for RA, assessed before and after a symptomatic COVID-19 infection. The main limitation of this study is the low incidence of seroconversion and symptom onset, despite the study population being genetically at risk for RA. Due to the limited number of cases, adjusted analyses were not feasible and restricted the statistical power of our analysis. Still, our results certainly do not suggest a major impact of SARS-CoV-2 infections on RA development. An additional limitation was the short follow-up after SARS-CoV-2 infection (mean of 11.6 month [6–15.6], with a maximum of 30 month). This limited duration may not have captured potential longer-term COVID-19 effects or potential delayed onset of RA-associated manifestations, which can develop over an extended period. Therefore, the study’s findings should be interpreted with caution regarding the long-term impact of SARS-CoV-2 infection on the development of RA-related outcomes. In addition, there are several other limitations to consider, including the possibility of underreporting or misclassification of the exposure and the lack of access to testing. Objective measures of SARS-CoV-2 infection, such as laboratory-confirmed cases, could help reduce bias associated with self-reporting, but the serology against SARS-CoV-2 also decrease over time may become falsely negative after an average follow-up of 1 year. The assessment of RA-associated clinical symptoms relied on self-reporting or subjective measures and criteria, which may have limitations in terms of accuracy and reliability. The non-randomized cohort study design introduced potential selection bias. In addition, since the study was conducted within a cohort of RA-FDRs, it is important to acknowledge that the generalizability of our findings to the broader population may be limited. More research is needed to determine whether the association suggested between SARS-CoV-2 infection and RA-associated autoimmunity or incident RA is coincidental or causal.

In conclusion, this analysis found no evidence of an increased risk of developing RA or RA-associated autoimmunity after a SARS-CoV-2 infection in susceptible individuals.

## Data availability statement

The raw data supporting the conclusions of this article will be made available by the authors, without undue reservation.

## Ethics statement

The studies involving human participants were reviewed and approved by Geneva Ethics Committee. The patients/participants provided their written informed consent to participate in this study.

## Author contributions

CL designed the study, performed the data analysis, participated in the interpretation of the results, designed, and wrote the manuscript. BG was involved in data management and the data analysis, participated in the interpretation of the results, and provided major revisions of the manuscript. OS was involved in data acquisition (follow-up visits, sampling). KL provided revisions of the manuscript. AF, principal investigator of the SCREEN-RA cohort, helped design the study, participated in the interpretation of the results, and provided major revisions of the manuscript. All authors contributed to the article and approved the submitted version.

## Funding

This work was supported by a grant from the Swiss National Science Foundation: no. 320030_192471/1.

## Conflict of interest

The authors declare that the research was conducted in the absence of any commercial or financial relationships that could be construed as a potential conflict of interest.

## Publisher’s note

All claims expressed in this article are solely those of the authors and do not necessarily represent those of their affiliated organizations, or those of the publisher, the editors and the reviewers. Any product that may be evaluated in this article, or claim that may be made by its manufacturer, is not guaranteed or endorsed by the publisher.
