# Supplementary material for: Brief report: can COVID-19 infection trigger rheumatoid arthritis-associated autoimmunity in individuals at risk for the disease? A nested cohort study
Source: Front Med (Lausanne). 2023 Jul 7;10:1201425. doi: 10.3389/fmed.2023.1201425 (PMC10361728; doi:10.3389/fmed.2023.1201425)
Supplement: Supplementary file 1 [file Table_1.DOCX]

**Table S1: Socio-demographic characteristics and serological status of RA-FDRs with self-reported data associated with Sars-CoV-2 vaccination.**

|  | | **Not vaccinated** | **Vaccinated** | **p** | **Missing (%)** |
| --- | --- | --- | --- | --- | --- |
| RA-FDRs (n) | | 58 | 173 |  |  |
| Gender = female (n (%)) | | 48 (82.8) | 141 (81.5) | 0.99 |  |
| Age (years; mean (SD)) | | 50.4 (12.7) | 55.6 (11.6) | <0.01 |  |
| BMI (mean (SD)) | | 24.2 (3.8) | 24.7 (4.5) | 0.42 |  |
| Share epitope (2 copies) (n (%)) | | 6 (10.3) | 36 (20.8) | 0.15 |  |
| Anti-CCP2 positive (n (%)) | | 1 (1.7) | 3 (1.7) | 0.05 | 17 (7.4) |
| Anti-CCP3 IgG positive (n (%)) | | 2 (3.4) | 1 (0.6) | <0.01 | 33 (14.3) |
| RF IgM positive (n (%)) | | 10 (17.2) | 29 (16.8) | 0.04 | 17 (7.4) |
| RF IgA positive (n (%)) | | 2 (3.4) | 9 (5.2) | 0.04 | 17 (7.4) |
| RA-associated symptoms (n (%)) | | 8 (13.8) | 32 (18.5) | 0.54 |  |
| Incident RA (n (%)) | | 1 (1.7) | 1 (0.6) | 1.00 |  |
| Self-reported data | Sars-CoV-2 infection | 29 (50) | 90 (52) | 0.79 | 60 (26.0) |
|  | Sars-CoV-2 test |  |  | 0.15 | 62 (26.8) |
|  | - PCR test (n (%)) | 38 (65.5) | 115 (66.5) |  |  |
|  | - Anti-S1 serology (n (%)) | 2 (3.4) | 12 (6.9) |  |  |
|  | Sars-CoV-2-associated symptoms (n (%)) | 11 (19) | 34 (19.7) | 0.95 | 112 (48.5) |
|  | Sars-CoV-2-associated hospitalisation (n (%)) | 2 (3.4) | 1 (0.8) | 0.24 | 180 (77.9) |
| Delay between sample 1 and sample 2 in years (median [IQR]) | | 4.2 [2.4-8.2] | 5.4 [3.0-8.4] | 0.12 |  |

All variables were defined at sample 2 (per/post-pandemic sample) collection; Anti-CCP2, anti-CCP3 IgG, RF IgM and RF IgA positivity: 1 time upper limit of normal (ULN); RA-associated symptoms: clinically suspected arthralgia (CSA) or presence of at least one swollen joint. RA = Rheumatoid Arthritis, RF= Rheumatoid Factor, anti-CCP = anti-Cyclic Citrullinated Peptide, FDR= First-Degree Relative, PCR: Polymerase Chain Reaction, S1= spike subunit 1.

**Table S2: Evolution of RA-associated autoantibodies and symptoms during SARS-CoV-2 pandemic in a subgroup of RA-FDRs who self-reported data associated with Sars-CoV-2 vaccination.**

|  | **Not vaccinated** | **Missing (%)** | **Vaccinated** | **Missing (%)** | **Crude RR**  **(95% CI)** |
| --- | --- | --- | --- | --- | --- |
| RA-FDRs (n) | 58 |  | 173 |  |  |
| **Primary outcomes** |  |  |  |  |  |
| Positive increase of anti-CCP2 ratio (≥1) (n (%)) | 0 (0.0) | 1 (1.7) | 2 (1.2) | 21 (12.1) | NA |
| Positive increase of anti-CCP3 IgG ratio (≥1) (n (%)) | 0 (0.0) | 39 (67.2) | 0 (0.0) | 112 (64.7) | 1.00 |
| Positive increase of RF IgM ratio (≥1) (n (%)) | 3 (5.2) | 19 (32.8) | 7 (4) | 54 (31.2) | 0.76 (0.21-2.82) |
| Positive increase of RF IgA ratio (≥1) (n (%)) | 0 (0.0) | 19 (32.8) | 1 (0.6) | 54 (31.2) | NA |
| **Secondary outcomes** |  |  |  |  |  |
| Becoming CCP2 positive (n (%)) | 0 (0.0) | 1 (1.7) | 2 (1.2) | 21 (12.1) | NA |
| Becoming anti-CCP3 IgG positive (n (%)) | 0 (0.0) | 39 (67.2) | 0 (0.0) | 112 (64.7) | 1.00 |
| Becoming RF IgM positive (n (%)) | 4 (6.9) | 19 (32.8) | 6 (3.5) | 54 (31.2) | 0.50 (0.15-1.68) |
| Becoming RF IgA positive (n (%)) | 0 (0.0) | 19 (32.8) | 2 (1.2) | 54 (31.2) | NA |
| Developing RA-associated symptoms (n (%)) | 5 (8.6) | 0 (0.0) | 24 (13.9) | 0 (0.0) | 1.61 (0.64-4.02) |
| RA development (n (%)) | 0 (0.0) | / | 0 (0.0) | / | 1.00 |

All variables were defined at sample 2 (per/post-pandemic sample) collection. Positive increase of anti-CCP2, anti-CCP3 IgG, RF IgM and RF IgA ratio: an increase beyond at least 1x the upper limit of the norm (≥1x ULN) between sample 2 and sample 1 (pre-pandemic sample); RA-associated symptoms: clinically suspected arthralgia (CSA) or presence of at least one swollen joint. RA = Rheumatoid Arthritis, RF= Rheumatoid Factor, anti-CCP = anti-Cyclic Citrullinated Peptide, FDR= First-Degree Relative, NA= Not applicable, RR= Relative Risk, CI=Confidence Intervals. A complete case analysis was used to determine crude RR.

**Table S3: Crude incidence rate of each outcome in SARS-CoV-2 infected and non-infected groups**

|  | **RA-FDRs with no self-reported Sars-CoV-2 infection** | **RA-FDRs with self-reported Sars-CoV-2 infection** |
| --- | --- | --- |
| **Primary outcomes** | **Crude IR (95% CI)** | **Crude IR (95% CI)** |
| Positive increase of anti-CCP2 ratio (≥1) (n (%)) | 29.2 (22.5-37.8)) | 5.80 (4.7-7.1) |
| Positive increase of anti-CCP3 IgG ratio (≥1) (n (%)) | 0 | 0 |
| Positive increase of RF IgM ratio (≥1) (n (%)) | 62.9 (46.5-85-1) | 24.7 (19.5-31.3) |
| Positive increase of RF IgA ratio (≥1) (n (%)) | 12.6 (9.3-17.0) | 8.3 (6.5-10.5) |
| **Secondary outcomes** |  |  |
| Becoming CCP2 positive (n (%)) | 9.7 (7.5-12.6) | 5.8 (4.8-7.2) |
| Becoming anti-CCP3 IgG positive (n (%)) | 0 | 0 |
| Becoming RF IgM positive (n (%)) | 63.7 (46.9-86.5) | 8.6 (6.7-10.9) |
| Becoming RF IgA positive (n (%)) | 0 | 8.3 (6.5-10.5) |
| Developing RA-associated symptoms (n (%)) | 102.6 (79.3-132.7) | 39.0 (32.3-47.0) |
| RA development (n (%)) | 0 | 0 |

All variables were defined at sample 2 (per/post-pandemic sample) collection. Positive increase of anti-CCP2, anti-CCP3 IgG, RF IgM and RF IgA ratio: an increase beyond at least 1x the upper limit of the norm (≥1x ULN) between sample 2 and sample 1 (pre-pandemic sample); RA-associated symptoms: clinically suspected arthralgia (CSA) or presence of at least one swollen joint. RA = Rheumatoid Arthritis, RF= Rheumatoid Factor, anti-CCP = anti-Cyclic Citrullinated Peptide, FDR= First-Degree Relative, IR: Incidence Rate per 1000 person-years; CI=Confidence Intervals. A complete case analysis was used to determine crude IR. The exposure time was defined as the duration between the onset of the SARS-CoV-2 outbreak in March 2020 and the date of sample 2 collection.

**Table S4: Crude incidence rate of each outcome in SARS-CoV-2 vaccinated and non-vaccinated groups**

|  | **Not vaccinated group** | **Vaccinated group** |
| --- | --- | --- |
| **Primary outcomes** | **Crude IR (95% CI)** | **Crude IR (95% CI)** |
| Positive increase of anti-CCP2 ratio (≥1) (n (%)) | 0 | 6.7 (5.7-7.8) |
| Positive increase of anti-CCP3 IgG ratio (≥1) (n (%)) | 0 | 0 |
| Positive increase of RF IgM ratio (≥1) (n (%)) | 55.3 (40.4-75-7) | 29.8 (24.5-35.7) |
| Positive increase of RF IgA ratio (≥1) (n (%)) | 0 | 4.3 (3.6-5.1) |
| **Secondary outcomes** |  |  |
| Becoming CCP2 positive (n (%)) | 0 | 6.7 (5.7-7.8) |
| Becoming anti-CCP3 IgG positive (n (%)) | 0 | 0 |
| Becoming RF IgM positive (n (%)) | 75.6 (55.0-103.9) | 26.6 (22.1-31.9) |
| Becoming RF IgA positive (n (%)) | 0 | 8.5 (7.1-10.2) |
| Developing RA-associated symptoms (n (%)) | 63.5 (49.1-82.1) | 68.7 (59.2-79.7) |
| RA development (n (%)) | 0 | 0 |

All variables were defined at sample 2 (per/post-pandemic sample) collection. Positive increase of anti-CCP2, anti-CCP3 IgG, RF IgM and RF IgA ratio: an increase beyond at least 1x the upper limit of the norm (≥1x ULN) between sample 2 and sample 1 (pre-pandemic sample); RA-associated symptoms: clinically suspected arthralgia (CSA) or presence of at least one swollen joint. RA = Rheumatoid Arthritis, RF= Rheumatoid Factor, anti-CCP = anti-Cyclic Citrullinated Peptide, FDR= First-Degree Relative, IR: Incidence Rate per 1000 person-years; CI=Confidence Intervals. A complete case analysis was used to determine crude IR. The exposure time was defined as the duration between the onset of the SARS-CoV-2 outbreak in March 2020 and the date of sample 2 collection.
